# Genetic mechanism for the loss of *PRAME* in B cell lymphomas

**DOI:** 10.1172/JCI160983

**Published:** 2022-07-15

**Authors:** Marek Mraz

**Affiliations:** 1 Molecular Medicine, CEITEC Masaryk University, Brno, Czech Republic.; 2 Department of Internal Medicine, Hematology and Oncology, University Hospital Brno and Faculty of Medicine, Masaryk University, Brno, Czech Republic.

**Keywords:** Immunology, Adaptive immunity, Immunoglobulins

## To the Editor:

**Figure 1:**
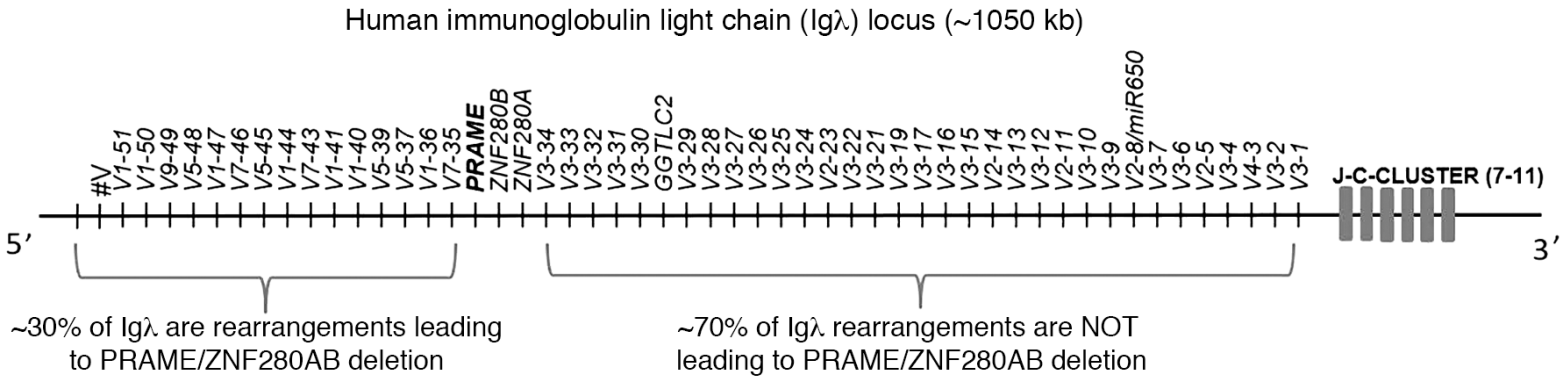
Schematic of the human Igλ locus organization and the location of the *PRAME* gene.

Takata et al. (1) reported that patients with diffuse large B cell lymphoma (DLBCL) relatively frequently (13% of patients) harbor a deletion at the 22q11.22 locus that involves the *PRAME* gene, and that *PRAME* loss is associated with poor outcomes and leads to cytotoxic T cell immune escape. The authors comment that “deletions...were located close to the Igλ gene.” I would like to bring to the attention of the authors and readers that the *PRAME* gene and neighboring *ZNF280A, ZNF280B*, and *GGTLC2* genes are located between variable (V) subgenes for the immunoglobulin lambda (Igλ) light chain (Figure 1). The *PRAME* deletion is inevitable when a B lymphocyte (normal or malignant) rearranges the Igλ locus and utilizes one of the many V subgenes located more distantly from the J-C region. It is known that approximately 30% to 40% of B lymphocytes express Igλ (~60%–70% express Igκ, since this locus for the Ig light chain is rearranged before Igλ). Therefore, it is not surprising that the loss of *PRAME* has been previously noted in multiple B cell malignancies, especially chronic lymphocytic leukemia (2–4). Takata et al. (1) observed that patients with *PRAME* deletions more often have an Igλ rearrangement, but they also report cases of DLBCL with a *PRAME* deletion and rearranged Igκ. However, it is not clear if in such cases the Igκ rearrangement was productive and what the status of the Igλ locus was. A defective allelic exclusion process might lead to Igκ and Igλ expression in one B cell. *PRAME* deletion associates with prognosis in DLBCL (1), but it should be considered that such a deletion could also be viewed as a surrogate marker for the use of one of the distal Igλ V subgenes (Figure 1), and it is known that Igλ usage associates with prognosis and B cell receptor (BCR) pathway deregulation in B cell malignancies (5).

In summary, loss of *PRAME* is an expected phenomena in a portion of normal or malignant B cells with Igλ rearrangement. It remains puzzling why in evolution *PRAME* has been placed between Igλ subgenes and why its expression is activated in DLBCL.

## Version 1 07/15/2022

Electronic publication
